# Desmosterol Incorporation Into Ram Sperm Membrane Before Cryopreservation Improves *in vitro* and *in vivo* Fertility

**DOI:** 10.3389/fcell.2021.660165

**Published:** 2021-06-24

**Authors:** María de las Mercedes Carro, Rafael R. A. Ramírez-Vasquez, Daniel A. Peñalva, Jorgelina Buschiazzo, Federico A. Hozbor

**Affiliations:** ^1^Instituto de Innovación para la Producción Agropecuaria y el Desarrollo Sostenible (IPADS Balcarce), Instituto Nacional de Tecnología Agropecuaria (INTA)—Consejo Nacional de Investigaciones Científicas y Técnicas (CONICET), Balcarce, Argentina; ^2^Instituto de Investigaciones Bioquímicas de Bahía Blanca (INIBIBB), Consejo Nacional de Investigaciones Científicas y Técnicas (CONICET), Universidad Nacional del Sur (UNS), Bahía Blanca, Argentina; ^3^Departamento de Biología, Bioquímica y Farmacia, Universidad Nacional del Sur (UNS), Bahía Blanca, Argentina

**Keywords:** cryopreservation, ovine sperm, cholesterol, desmosterol, artificial insemination

## Abstract

Pregnancy rates in ewes are markedly low after cervical insemination with frozen-thawed sperm. Sensitivity of ram sperm to freeze-thawing is related to the lipid composition of the membrane, particularly to its low sterol content. Recently, we proved that sterol content of ram sperm can be increased by treatment with methyl-β-cyclodextrin-sterol complexes and we provided mechanistic based evidence on the differential behavior of cholesterol and desmosterol in the ram sperm membrane. In the present study, we evaluated the role of increasing cholesterol and desmosterol content of ram sperm before cryopreservation, on the extent and distribution of sterols, cryocapacitation status, acrosome integrity, DNA damage associated with apoptosis and fertility competence *in vitro* and *in vivo* of post-thawed sperm. After freeze-thawing, similar levels of sterol content were evidenced in control sperm cells and in those pre-incubated with either cholesterol or desmosterol. Still, moderately higher levels of sterols were registered in treated sperm compared to the control, indicating no physiological excess of sterols after thawing or sterol losses that exceed the control. Live cell imaging of fluorescent cholesterol evidenced the presence of sperm sub-populations differentially affected by freeze-thawing. Similar unimodal frequency profiles were observed between sterol-enriched groups, while the control exhibited a sub-population of sperm compatible with low sterol content. Tyrosine phosphorylation was significantly lower when ram sperm incorporated cholesterol compared to the control. No difference in this capacitation parameter was found between the latter and desmosterol-enriched sperm. The percentage of sperm with damaged acrosomes post-thawing, assessed by a fluorescent lectin, was reduced in sperm that incorporated sterols before freezing, irrespective of the sterol class. These results suggest that sterols exert a stabilizing effect on the acrosome. No differences were found in levels of apoptotic DNA fragmentation among experimental groups. As to fertility trials, desmosterol-enriched sperm gave rise to higher rates of *in vitro* activated oocytes by heterologous fertilization and to significantly lower pregnancy loss *in vivo*. Our research provides new insights on sterol incorporation into ram sperm prior to cryopreservation, in particular on the additional benefit of incorporating desmosterol as a strategy to improve fertility outcome.

## Introduction

Artificial Insemination (AI) is an essential tool for animal production industry, enabling the introduction of valuable reproductive and productive genetic backgrounds into breeding programs. Long-term cryopreservation of the male gamete was a major contribution to the development of AI for many species. However, pregnancy rates in sheep inseminated *via* cervical with frozen-thawed sperm are markedly low, and therefore not feasible for commercial breeding programs (Alvarez et al., 2019). In this species, the complex anatomical structure of the cervix prevents intrauterine deposition of semen by routine methods, having to resort to laparoscopic intrauterine insemination. Cervical insemination is less invasive and has a lower ethical and practical cost than laparoscopic procedures. However, fertilization rates obtained with this method rarely exceed 40%, limiting the commercial use of cryopreserved ram sperm (Alvarez et al., 2019). The high sensitivity of ram sperm to cryopreservation reduces their survival and transport in the female reproductive tract (Gillan and Maxwell, 1999; Robinson et al., 2011) highlighting the need for improved semen freezing methods in this species.

Post-thaw sperm quality is quite different among animal species as a result of their different sensitivity to freezing and thawing (Holt, 2000). The low survival rate of cryopreserved sperm is associated with structural and functional alterations such as plasma membrane functionality loss (Sieme et al., 2015), cytoskeleton protein damage (McClean et al., 2007), acrosomal detachment or rupture (Cross and Hanks, 1991; Okabe, 2016), DNA damage (Valcarce et al., 2013), and mitochondrial impairment (Treulen et al., 2018). The low sterol/phospholipid ratio of ram sperm membranes has been identified as one of the most significant factors determining their low tolerance to cold temperatures (Mocé et al., 2010a). Sterols in mammalian sperm membranes are mainly represented by cholesterol and to a lesser extent by its immediate precursor, desmosterol, a cholesterol isomer with an extra double bond that diminishes its ordering potential in lipid bilayers (Vainio et al., 2006; Keber et al., 2013). Along with the reduced sterol content, ovine sperm have a lower content of desmosterol than sperm from other species, which is converted to cholesterol during sperm epididymal transit and maturation (Parks and Hammerstedt, 1985). While the efflux of sterols has a physiological role on sperm capacitation (Cross, 2003), sterol loss occurred during cryopreservation has a significant impact on sperm viability by inducing a premature capacitation-like state that shortens the life span of the spermatozoa (Mocé et al., 2010a).

Temperature decrease induces lateral lipid segregation, altering the lipid-lipid and lipid-protein interactions in the plasma membrane, compromising its functionality and integrity (Leahy and Gadella, 2011). It has been proposed that decreasing these lipid phase transitions during cooling is a strategy to reduce the sensitivity of sperm membranes to cryopreservation (Sieme et al., 2015). Sterols are known to modulate phase behavior of lipid membranes by increasing the order of acyl chains while maintaining lipid translational fluidity. High sterol content in sperm membranes during cooling keeps the phospholipid bilayer in a lamellar arrangement for a wider temperature range, reducing membrane phase transitions. Moreover, increasing sterol content of sperm before cryopreservation through incubation with sterols complexed with methyl-β-cyclodextrin (MβCD), a cyclic oligosaccharide used as a sterol donor, has been shown to reduce cryodamage in several species (Purdy and Graham, 2004; Moore et al., 2005; Müller et al., 2008), including the ram (Mocé et al., 2010b; Purdy et al., 2010; Motamedi-Mojdehi et al., 2014; Salmon et al., 2017). However, when AI was performed after MβCD-cholesterol treatment, no increase in pregnancy rates was reported (Purdy et al., 2010; Salmon et al., 2017). It is important to point out that many of these studies were performed in the presence of lipids from cryopreservation semen extenders containing egg yolk or skim milk.

Recently, we quantified the extent of cholesterol and desmosterol incorporation into ram sperm by incubation with MβCD-sterol complexes prior to cryopreservation in combination with a sterol-free extender (Carro et al., 2020). Through a biophysical approach, we demonstrated that both sterols similarly increased membrane lipid order according to total sterol increase, which was different depending on the MβCD-sterol concentration used. Treatment with 10 mM MβCD complexed either with cholesterol or desmosterol did not disturb lateral organization of the plasma membrane, improving its tolerance to osmotic stress and sperm total motility after refrigeration. Interestingly, when classical sperm quality parameters were evaluated after freeze-thawing, sterols showed a differential protective effect compared to non-treated sperm. While treatment with 10 mM MβCD-cholesterol increased sperm motility, membrane integrity and tolerance to osmotic stress, incorporation of desmosterol revealed an increased ability of ram sperm to withstand osmotic stress.

Based on these promising results and considering fertility outcomes of cryopreserved ram sperm as a primarily goal of our studies, we aimed at deepen our understanding of the role of cholesterol and desmosterol in ram sperm functionality and fertility. In the present study, we compared the extent and distribution of sterols, cryocapacitation status, acrosome integrity, DNA damage associated with apoptosis and *in vitro* and *in vivo* fertility competence of frozen-thawed ram sperm treated with cholesterol or desmosterol before cryopreservation.

## Materials and Methods

### Chemicals and Reagents

Cholesterol, MβCD and FITC-Concanavalin A (ConA) were purchased from Sigma Chemical Company (St. Louis, MO, United States). Desmosterol and BODIPY-Cholesterol (BPY-Chol) were purchased from Avanti Polar Lipids (Alabaster, AL, United States). All solvents used in this study were HPLC grade (JT Baker, United States; UVE, Argentina).

### Preparation of Methyl-β-Cyclodextrin-Sterol Complexes

MβCD-sterol complexes were prepared as reported previously (Carro et al., 2020). Briefly, a volume of cholesterol or desmosterol from a stock solution (10 μg/μL) in chloroform:methanol 1:1 (v/v) was added to a glass tube and the solvent was evaporated under a gentle stream of nitrogen for 1 h. A volume of 0.5 M stock solution of MβCD in Tris-citric acid buffer (300 mM Tris, pH 7.4, 95 mM citric acid) was subsequently added to the dried material (MβCD:sterol, 4:1 molar ratio) to reach the concentration of 40 mM MβCD:sterol. The mixture was clarified by vigorous mixing, subjected to bath sonication for 5 min and incubated in a rotating water bath at 37°C overnight. Before using the solution, it was centrifuged at 2000 × g for 10 min to remove excess of sterol crystals.

### Semen Collection and Processing

Animals used in this study were handled in strict accordance with Good Animal Welfare Practices approved by the Institutional Committee for the Care and Use of Experimental Animals (CICUAE-Resolution 046/2016). Twenty mature Texel rams were kept on a ray grass, fescue and white clover pasture (37° 450 S, 58° 180 W), with water *ad libitum.* Semen was obtained using an artificial vagina during the breeding season in the southern hemisphere (March-June) following a routine collection of twice a week with at least 2 days of sexual abstinence. Only semen samples with mass motility ≥4 were processed. In order to avoid individual effects, semen from four randomly selected rams were pooled in each collection date. Pooled semen was divided into aliquots to conform each experimental group. The number of repetitions (number of pools) for each evaluation is indicated in the figure legend. Ejaculates were placed in a water bath at 32°C until processing. Sperm concentration was assessed in a Neubauer hemocytometer under light microscopy. Subsequently, semen was pooled with each male contributing the same number of spermatozoa and concentration was adjusted to 3,000 × 10^6^ sperm/mL in pre-warmed (32°C) Tris-citric acid buffer. The pool was divided into three aliquots to conform each experimental group: Control (non-treated), Chol (pre-treated with 10 mM MβCD-cholesterol) and Des (pre-treated with 10 mM MβCD-desmosterol) and incubated for 15 min with 0 (control) or 10 mM MβCD-sterol. In order to remove MβCD molecules after incubation, spermatozoa were washed by centrifugation (1000 × g, 5 min) with pre-warmed extender or Tris-citric acid buffer followed by sperm cryopreservation.

### Semen Cryopreservation

Semen pre-incubated with Tris-citric acid buffer (control) or MβCD-sterol (treatment) was diluted in three steps with a soy lecithin-based commercial extender (Andromed© Minitüb, Germany) to a final concentration of 400 × 10^6^ sperm/mL and packaged into 0.25 mL straws (Minitüb, Germany). Temperature was progressively decreased from 32 to 5°C with a 0.1°C/min rate in a water bath and equilibrated for 2 h at 5°C. Straws were frozen in liquid nitrogen vapors (4 cm above the liquid level) for 10 min and finally stored in liquid nitrogen tanks. Thawing was performed by immersion of the straws in a water bath at 37°C for 30 s.

### Quantification of Sperm Sterols

Spermatozoa were washed by centrifugation in citrate-EDTA buffer (35.5 mM sodium citrate, pH 7.4, 2.5 mM EDTA) at 2,500 × g for 10 min. Lipids were extracted with chloroform:methanol (1:2, v/v) according to Bligh and Dyer (1959). Total sterols were quantified by an analytical method using a commercially available enzymatic assay (Colestat Wiener Lab, Rosario, Argentina). An aliquot from the lipid extract, equivalent to 140 × 10^6^ spermatozoa, was placed into a glass tube and dried under a nitrogen stream. The dried extract was resuspended in isopropyl alcohol and vigorously mixed for 1 min. In this procedure, 1 mL of Working Reagent was combined with 100 μL of isopropyl alcohol extract and incubated at room temperature (25°C) for 30 min. The absorbance was measured in a spectrophotometer at 505 nm and compared to a standard curve.

Cholesterol and desmosterol were quantified in cryopreserved ram sperm by reverse high-pressure liquid chromatography (HPLC). Lipid extracts from spermatozoa were spotted on thin-layer chromatography (TLC) plates (500 μM, silica gel G) along with commercial standards. The sterol fraction was resolved using hexane:ether:ammonia (45:65:1, v/v). Lipid bands were located under ultraviolet light after spraying the TLC plates with 2,7-dichlorofluorescein in methanol and exposing them to ammonia vapor. The sterol fraction was eluted with chloroform:methanol:water (5:5:1, v/v) and partitioned by the addition of 0.8 volumes of water. Reverse phase HPLC was performed at 40°C with a C18 HPLC column (Agilent Technologies, United States; 100 cm × 4.6 mm, 3.5 μM) equilibrated with methanol (100%) at a flow rate of 1 mL/min. A standard curve was done by monitoring absorbance of increasing concentrations of commercial standards at 205 nm. Peaks of each sterol were identified and plotted against the corresponding concentration to create the calibration curve.

### Imaging of BODIPY-Cholesterol in Living Sperm

A stock solution (1 mM) of BPY-Chol was prepared in ethanol and stored in a dark glass tube at −20°C protected from light. A sperm suspension was diluted to a final concentration of 100 × 10^6^ spermatozoa/mL in IVF-SOF medium supplemented with 1 μM of BPY-Chol and 0.5 μM propidium iodide and incubated at 38.5°C under 5% CO_2_ in humidified air for 1 h in the dark. After incubation, 20 μL of the sperm suspension was placed for 10 min on a slide previously coated with poly-L-lysine. Adhered cells were washed with IVF-SOF medium and observed using an epifluorescence inverted microscope (Nikon TE-300; Nikon, Tokyo, Japan). The fluorescent dye BPY-Chol was excited at 450–490 nm and digital photographs were taken with a DSfi1 camera (Nikon, Tokyo, Japan) connected to the microscope. Fluorescence intensity of sperm from randomly selected microscope fields was measured using the software Fiji (Schindelin et al., 2012) in background corrected images. A mask was created to locate each sperm-associated fluorescence and measure the mean gray value (MGV) from randomly selected fields at 40× magnification. At least 200 spermatozoa were counted in each image. To study differences in fluorescence intensity among spermatozoa within a population of cells, relative frequency histograms were performed by grouping MGV in 5-arbitrary fluorescence units (AFU) ranges.

### Determination of Tyrosine Phosphorylation Levels by Western Blot

Sperm proteins were isolated following the protocol from Romarowski et al. (2015). Briefly, 10 × 10^6^ spermatozoa from each group were washed by centrifugation in citrate-EDTA buffer at 800 × g for 3 min. The supernatant was removed and the pellet was resuspended in 100 μL of 62.5 mM Tris-HCl pH 6.8, 2% sodium dodecylsulfate (SDS) with 10% glycerol and boiled for 4 min. After centrifugation at 16000 × g for 5 min, supernatant was recovered, supplemented with 5% β-mercaptoethanol and boiled for 5 min once again. Bromophenol blue was added to reach a final concentration of 0.0005% and protein extracts were stored at −80°C until analysis by Western blot.

Protein extracts corresponding to 5 × 10^6^ spermatozoa were separated by SDS-polyacrylamide gel electrophoresis (PAGE) (12.5% acrylamide) (Laemmli, 1970) and transferred to PVDF membranes (Immobilon-P, Millipore, Bedford, MA). Membranes were blocked overnight in TBS-T (20 mM Tris-HCl, pH 7.5, 150 mM NaCl, 0.05% Tween-20) supplemented with 0.5% BSA and 1% non-fat dry milk at 4°C followed by incubation with a mouse anti-phosphotyrosine antibody (pTyr) conjugated to HRP (ab16389, Abcam United Kingdom) diluted 1:2500 in TBS-T supplemented with 0.5% BSA for 4 h at room temperature. After pTyr detection, membranes were stripped with gentle shaking in stripping buffer (62.5 mM Tris-HCl, pH 6.8, 2% SDS, 100 mM β-mercaptoethanol) for 15 min at 55°C. Membranes were then blocked and incubated with a monoclonal mouse anti α-Tubulin antibody (α-Tub, T9026, Millipore Sigma, United States) diluted (1:5000) in 1% non-fat dry milk in TBS-T for 1 h at room temperature followed by secondary antibody incubation (Abcam, United Kingdom) diluted 1:10000 in TBS-T supplemented with 1% non-fat milk for 1 h at room temperature. Immunoreactive bands were revealed by chemiluminescence using the Bio-lumina kit (*Productos Biológicos*, Argentina) and autoradiographic films (General Electrics, United States). Films were scanned and quantified by densitometry with Fiji software (Schindelin et al., 2012).

### Acrosome Status

Acrosome status integrity of frozen-thawed ram sperm was assessed by incubation with a fluorescent (FITC) Concanavalin A (ConA) (Ozaki et al., 2002). Sperm suspensions (10 × 10^6^ cells) were washed by centrifugation at 800 × g for 3 min in PBS and fixed overnight at 4°C in 4% formaldehyde in PBS (pH 7.4). Cells were pelleted again, resuspended in 20 μL of PBS and smeared onto a slide previously coated with poly-L-lysine for 20 min. Slides were then washed with PBS and stained with ConA 100 μg/mL in PBS solution in the dark for 20 min at room temperature. After incubation, slides were washed again with PBS and covered with a coverslip. Spermatozoa were observed using an epifluorescence inverted microscope (Nikon TE-300; Nikon, Tokyo, Japan). The fluorescent lectin ConA was excited at 450–490 nm and digital photographs were taken with a DSfi1 camera (Nikon, Tokyo, Japan) connected to the microscope. Sperm with non-intact acrosome showed bright fluorescence in the acrosomal region (ConA+), while intact acrosome did not show fluorescence (ConA−). At least 200 spermatozoa were examined and the percentage of spermatozoa ConA+ was calculated for each group. The number of ConA+ sperm was normalized by the number of live sperm on each condition evaluated by the eosin-nigrosin exclusion test (Björndahl et al., 2003).

### Detection of Apoptotic DNA Fragmentation

Apoptotic DNA fragmentation induced by cryopreservation was analyzed using the *in situ* Cell Death Detection Kit with fluorescein (Roche Diagnostics GmbH, Mannheim, Germany) that detects DNA breaks associated with apoptosis. This kit is based in the terminal deoxynucleotidyl transferase-mediated fluorescein dUTP nick-end labeling reaction (TUNEL) of free 3′-OH *termini* by terminal deoxynucleotidyl transferase (TdT). Thawed sperm were washed twice by centrifugation in 6 mL PBS supplemented with BSA (1 mg/mL) for 5 min at 200 × g. A drop of re-suspended spermatozoa was smeared on a glass slide, air dried and fixed with 4% formaldehyde in PBS for 60 min at room temperature and stored at 4°C until use. Slides were washed in PBS and cells were permeabilized with freshly prepared 0.1% Triton X-100 in 0.1% sodium citrate for 5 min on ice. Slides were rinsed twice with PBS, and cells were incubated with the TUNEL reaction mixture in the dark for 60 min at 37°C in a humidified chamber. After this period, slides were washed three times with PBS and analyzed by fluorescence microscopy using an excitation wavelength of 488 nm (Nikon TE-300; Nikon, Tokyo, Japan). Negative (omitting TdT from the reaction mixture) and positive (using DNase I, RQ1 Promega for 10 min at room temperature) controls were included in each trial. At least 200 sperm in each experimental group were evaluated to determine the percentage of TUNEL-positive sperm (green fluorescence in the head).

### Heterologous *in vitro* Fertilization

Heterologous *in vitro* fertilization (IVF) using bovine oocytes was employed to assess the fertility of thawed ram sperm samples according to García-Alvarez et al. (2009) with some modifications.

#### Oocyte Collection and *in vitro* Maturation

Bovine ovaries from cycling beef heifers (*Bos taurus taurus*) were collected from local slaughterhouses and transported within 60–90 min in a thermic container to the laboratory. *Cumulus*-oocyte complexes (COCs) were aspirated from follicles ranging from 2 to 8 mm in diameter by a vacuum system. COCs with homogeneous ooplasm and more than four complete layers of *cumulus* cells, corresponding to grades 1 and 2 according to de Loos et al. (1989) were selected under a stereomicroscope and washed 3 times in M199 supplemented with 0.5% Hepes (w/v). Selected COCs were incubated in four-well culture plates (NUNC, Thermo Fisher Scientific, Loughborough, Leicestershire, United Kingdom) in groups of 60 per well, with 400 μL of M199 medium (Sigma-Aldrich, United States), supplemented with 0.1 mg/ml L-glutamine, 2.2 mg/ml sodium bicarbonate and 0.1 IU/mL rhFSH (Gonal F-75, Serono, United Kingdom) for 22 h at 38.5°C under 5% CO_2_ in humidified air.

#### *In vitro* Fertilization

*Cumulus* cells were partially removed from matured bovine oocytes by gently pipetting in M199-Hepes and oocytes were placed in four-well culture plates containing 400 μL of fertilization medium (SOF supplemented with 10% of estrous sheep serum and 40 μg/mL gentamycin). Thawed ram sperm were selected on a Percoll^®^ (Sigma-Aldrich, United States) discontinuous density gradient (45/90%) and incubated for 10 min in the fertilization medium. Thawed sperm from each experimental group were co-incubated with the oocytes at a final concentration of 2.5 × 10^6^ sperm/mL per well at 38.5°C in 5% CO_2_ in humidified air for 48 h.

To assess IVF rates, oocytes were fixed in 2% glutaraldehyde in M199-Hepes for 10 min and stained with 5 μM bisBenzimide Hoechst 33342 for 20 min at room temperature. Oocytes were mounted on a slide with glycerol, covered with a coverslip and observed under an epifluorescence microscope (Nikon TE-300; Nikon, Tokyo, Japan) using excitation wavelength of 380 nm. Heterologous IVF rates were determined as the percentage of activated oocytes (presence of *pronuclei* or cell cleavage). Experimental groups were defined as matured oocytes co-incubated with frozen-thawed sperm non-treated (Control), pre-treated with 10 mM MβCD-Cholesterol (Chol) and pre-treated with 10 mM MβCD-Desmosterol (Des). Five heterologous IVF experiments were performed in total and the number of oocytes for each experimental condition was indicated in the figure legend.

### *In vivo* fertility

To assess the effect of increasing sperm cholesterol or desmosterol content before freezing and thawing on *in vivo* fertility, we performed a cervical AI at fixed-time during the breeding season. For this purpose, 120 Texel ewes were randomly inseminated with frozen-thawed sperm from the three experimental groups (Control, Chol, Des) and pregnancy rate and lambing were recorded.

#### Estrous Synchronization

Synchronization of estrous was conducted by intravaginal sponges impregnated with medroxyprogesterone release (Progespon^®^, Syntex SA, Argentina, 60 mg per dose) and treated with oxytetracycline (Terramicina 100^®^, Zoetis, Argentina) for a period of 7 days. At sponge removal time, ewes were treated with equine chorionic gonadotropic (Novormon 5000^®^, Syntex, Argentina, 300 IU, im), and Cloprostenol (Ciclase^®^DL, 75 mg, im).

#### Artificial Insemination and Fertility Rates

Artificial Insemination was performed at fixed time, 48–50 h after withdrawal of intravaginal device. A dose of 100 × 10^6^ frozen-thawed sperm (0.2 mL volume) was placed between the first and second cervical rings. Early pregnancy was determined by transrectal ultrasound (Aloka SSD 500, Hitachi, Japan) 30 days after AI and lambing was registered for each ewe between days 140 and 160 after AI procedure.

### Statistical Analysis

Statistical analysis was carried out using InfoStat software (Di Rienzo et al., 2014). Sterol content and BPY-Chol fluorescence intensity were analyzed through analysis of variance (ANOVA), followed by *post hoc* test analysis of multiple comparisons by Fisher’s Least Significant Difference (LSD Fisher). Shapiro-Wilk and Levene tests were carried out to verify normal distribution and homogeneity of variances, respectively. Tyrosine phosphorylation (densitometry) was analyzed by non-parametric Kruskall-Wallis test. Percentages of acrosome status, sterol composition, TUNEL-positive sperm and *in vitro* fertility were compared using Generalized Linear Mixed Models (GLMM) with Binomial family, *logit* link function and LSD Fisher contrast. *In vivo* fertility rates were compared using the Chi-square test for homogeneity. Differences were considered significant at *p* < 0.05.

## Results

### Quantification and Distribution of Sterols in Cryopreserved Ram Sperm

**FIGURE 1:**
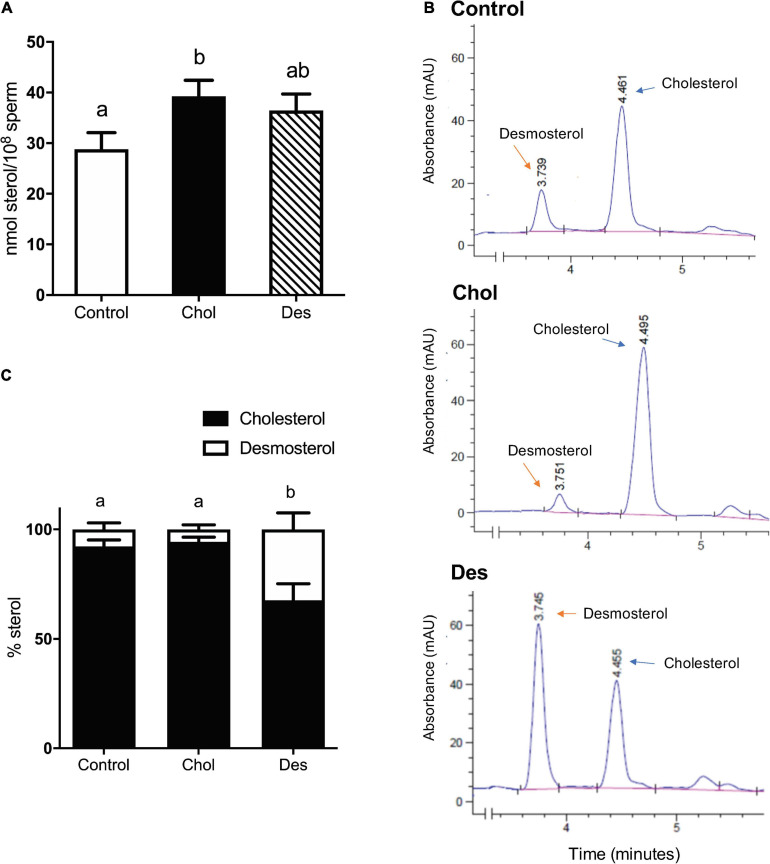
Quantification of cholesterol and desmosterol in cryopreserved ram sperm pretreated with 10 mM MβCD-Chol (Chol) -Des (Des). **(A)** Effect of 10 mM MβCD-Chol or -Des pretreatment on total sterol content of frozen-thawed ram sperm. Results are expressed as nmol of sterols per 100 million sperm and represent mean values ± SEM of 7 (control y MβCD-Des) and 10 (MβCD-Chol) independent experiments. **(B)** Identification of cholesterol and desmosterol by HPLC in the ovine sperm sterol fraction of Control, Chol and Des groups. The peaks correspond to each sterol with the indicated retention times. **(C)** Quantification of cholesterol and desmosterol by HPLC in each experimental group. The results are expressed as percentage of sterols and represent the mean values ± SD of three independent measurements. Sterol content (%) among the experimental groups was compared by Generalized Linear Mixed Models (GLMM) with binomial distribution. The different letters (a, b) indicate significant differences (*p* < 0.05). Data were analyzed by ANOVA and mean values were compared using the *post hoc* test LSD of Fisher. Different letters (a, b) indicate significant differences (*p* < 0.05).

To study the effect of increasing ram sperm sterol content on sperm functionality and fertility after cryopreservation, sperm were treated with 10 mM MβCD complexed either with cholesterol or desmosterol and subsequently frozen following a stepwise temperature decrease. After thawing, total sterol content was quantified from sperm lipid extracts with an enzymatic assay (Figure 1A) and the fraction corresponding to free sterols was resolved by reverse HPLC to assess the percentage distribution of free sterol classes in each group (Figure 1B). Sperm that incorporated cholesterol before freezing (Chol group) showed increased total sterol content compared to the control (Figure 1A). Nevertheless, the relative cholesterol and desmosterol percentage distribution within this group did not differ from the control, cholesterol being the main sterol (>93% of total sterols) in both experimental conditions (Figure 1C). On the contrary, sperm that incorporated desmosterol before cryopreservation (Des group) did not show a significant difference in total sterol content compared to the control or Chol group after thawing (Figure 1A). However, desmosterol content in this group was about five times higher (∼35% of total sterols) than the control or Chol group (Figure 1C).

### Comparing Sterol Behavior in Cryopreserved Sperm by *in situ* Imaging of BODIPY-Cholesterol

**FIGURE 2:**
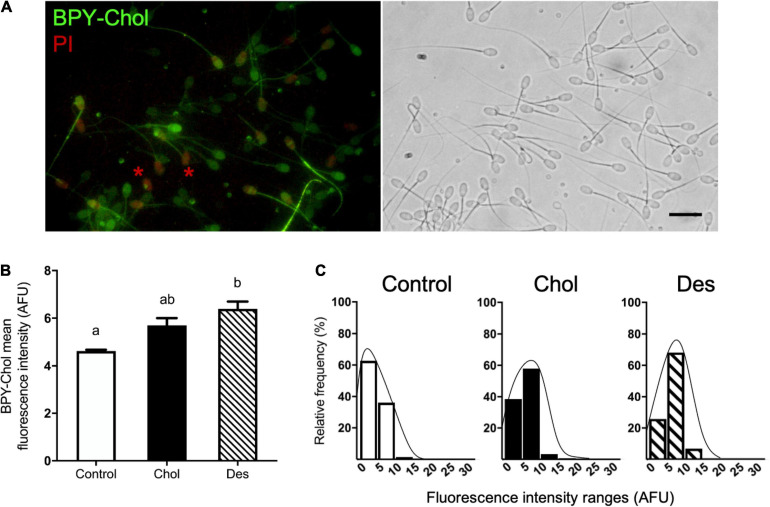
Sterol distribution and content in cryopreserved ram spermatozoa estimated by BPY-Chol labeling of living cells. **(A)** Representative fluorescence image of BPY-Chol and PI in ram sperm with bright field on the right, scale bar represents 20 μm. Asterisks indicate representative PI-positive stained sperm. **(B)** Sterol content of cryopreserved ovine spermatozoa after cholesterol (Chol) or desmosterol (Des) incorporation through treatment with 10 mM MβCD-sterol complexes. Results are expressed as arbitrary fluorescence units (AFU) and represent mean values ± SEM (*N* = 5). Fluorescence measurements were automatized using masks in Fiji software. A total of 692, 451, and 864 cells were quantified for the control, Chol and Des groups, respectively. Data were analyzed by ANOVA and mean values were compared using the *post hoc* Bonferroni test. Different letters (a, b) indicate significant differences (*p* < 0.05). **(C)** Histograms of the relative frequencies of BPY-Chol mean fluorescence intensity measured per spermatozoa and grouped in ranges of 5 AFU for each experimental group. Lines estimate unimodal histogram distribution.

To further investigate the sterol distribution and content of cholesterol and desmosterol in treated and non-treated sperm after cryopreservation, we incubated sperm with BPY-Chol (Figure 2A). Sperm that incorporated desmosterol before cryopreservation showed higher BPY-Chol fluorescence intensity levels with respect to the other experimental groups (Figure 2B). No statistical difference was found in BPY-Chol fluorescence intensity in sperm that incorporated cholesterol before freezing with respect to the control and Des group. As expected, living sperm incubated with BPY-Chol displayed differences in fluorescence intensities among cells, evidenced in fluorescence frequency histograms (Figure 2C). The presence of different mean fluorescence intensities per cell is compatible with the existence of different sterol content among cells within the same sperm population. All experimental groups showed a unimodal frequency distribution. While the majority of the cells in the control group displayed fluorescence intensities of 0–5 AFU, the most frequent values for pretreated sperm with MβCD-sterol ranged from 5 to 10 AFU, regardless of the type of sterol incorporated before freezing.

### Effects of Sterol Incorporation on Tyrosine Phosphorylation Levels and Acrosome Integrity After Freeze-Thawing

**FIGURE 3:**
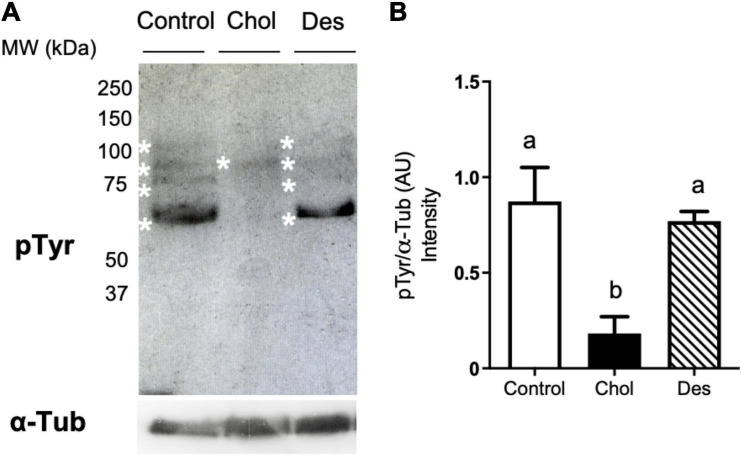
Identification of tyrosine phosphorylated proteins in cryopreserved ram sperm pre-treated with 10 mM MβCD-Chol (Chol) or -Des (Des). Proteins were resolved by SDS-PAGE (12.5%), blotted using an anti-phosphotyrosine (pTyr) antibody and immunoreactive bands were revealed by chemiluminescence. **(A)** Representative set of the experimental groups showing pTyr detection. Asterisks indicate position of the different phospho-proteins. Each lane corresponds to 5 × 10^6^ spermatozoa. Constitutive α-Tubulin (α-Tub) was used as loading control (lower panel). **(B)** Quantification of total intensity of immunoreactive bands per lane measured by densitometry (Fiji software) and expressed as arbitrary units (AU). Bars represent mean values ± SEM (*N* = 4). Data were analyzed by Kruskal-Wallis test. Different letters (a, b) indicate significant differences (*p* < 0.05).

To study the effect of cholesterol or desmosterol incorporation into ram sperm prior to freezing on the cryocapacitation status after thawing, premature activation of capacitation-like signaling induced by cryopreservation was evaluated through the analysis of tyrosine phosphorylation (Figure 3A). When ram sperm incorporated cholesterol prior to freezing, tyrosine phosphorylation levels of frozen-thawed sperm were significantly lower compared to the control (Figure 3B). On the other hand, sperm that previously incorporated desmosterol did not show a significant difference in this capacitation parameter compared to the control.

**FIGURE 4:**
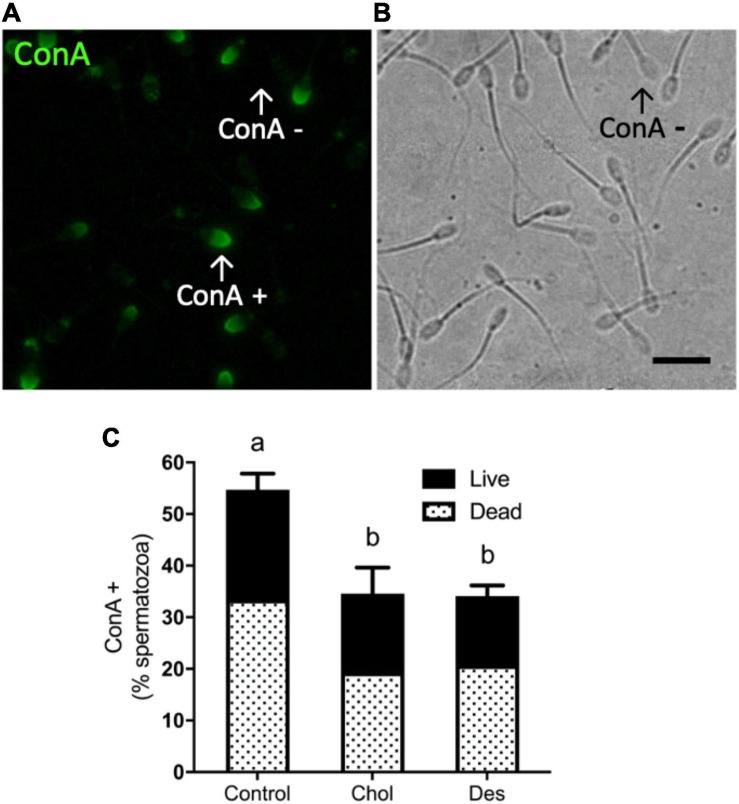
Effect of sterol incorporation on acrosome integrity after cryopreservation **(A)** Representative fluorescence image of cryopreserved ram spermatozoa incubated with FITC-Concanavalin A (ConA) showing stained cells (ConA+). **(B)** Bright field image. Scale bar represents 20 μm. Arrows indicate ConA + and ConA− spermatic cells in counterpart images. **(C)** Percentage of ConA + ram spermatozoa after thawing showing the normalized percentages of live/dead spermatozoa. Stacked bars represent the mean values ± SEM (*N* = 5). Data were analyzed using Generalized Linear Mixed Models (GLMM) with Binomial family. The different letters (a, b) indicate significant differences (*p* < 0.05).

In order to evaluate the effect of the incorporation of sterols prior to freezing on the acrosome integrity, spermatozoa were incubated with the fluorescent lectin ConA. Figure 4A shows the binding of ConA to acrosomal glycoproteins as a consequence of the exposure of the internal acrosome membrane during acrosome reaction, or due to acrosome integrity loss (Figures 4A,B). Treatment with MβCD-Chol or -Des prior to freezing significantly decreased (∼20%) the percentage of ConA positive sperm after thawing compared to the control, indicating a higher percentage of intact acrosomes in sperm that incorporated either cholesterol or desmosterol (Figure 4C). No significant difference was observed between treatments with the different sterols.

### Assessing DNA Damage Associated to Apoptosis in Cryopreserved Sperm

**FIGURE 5:**
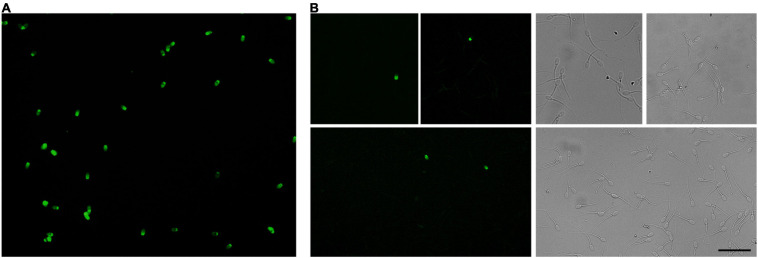
Apoptosis associated DNA fragmentation in cryopreserved ram sperm. DNA breaks in thawed spermatozoa were labeled using the terminal deoxynucleotidyl transferase-mediated dUTP nick-end labeling reaction (TUNEL). TUNEL-positive ram spermatozoa show green fluorescence in the head. **(A)** Positive control achieved by DNase I treatment with all spermatozoa showing green fluorescence. **(B)** Representative TUNEL-positive sperm from experimental groups on the left panels and respective bright fields on the right. Scale bar represents 40 μm.

To evaluate differences in DNA fragmentation induced by freezing and thawing of ram sperm, we performed TUNEL analysis. After labeling of DNA strand breaks by TdT (Figure 5), we observed a low level of DNA fragmentation (0.8–1.5% TUNEL-positive cells) with no significant differences among experimental groups (Table 1).

**TABLE 1:** Percentages of TUNEL-positive ram sperm after thawing.

Experimental groups	Total sperm (N)	TUNEL-positive (% ± SEM)
Control	762	1.48 ± 0.89^a^
Chol	728	0.96 ± 0.21^a^
Des	756	0.77 ± 0.72^a^

*DNA fragmentation was detected by the terminal deoxynucleotidyl transferase-mediated fluorescein dUTP nick-end labeling reaction (TUNEL) of free 3′-OH termini by terminal deoxynucleotidyl transferase (TdT). Data represent the mean values ± SEM (N = 3) and were analyzed using Generalized Linear Mixed Models (GLMM) with Binomial family. No differences were found among experimental groups (p > 0.05). ^*a*^ Means with the same letter are not significantly different.*

### *In vitro* Fertilizing Competence of Cryopreserved Sperm

**FIGURE 6:**
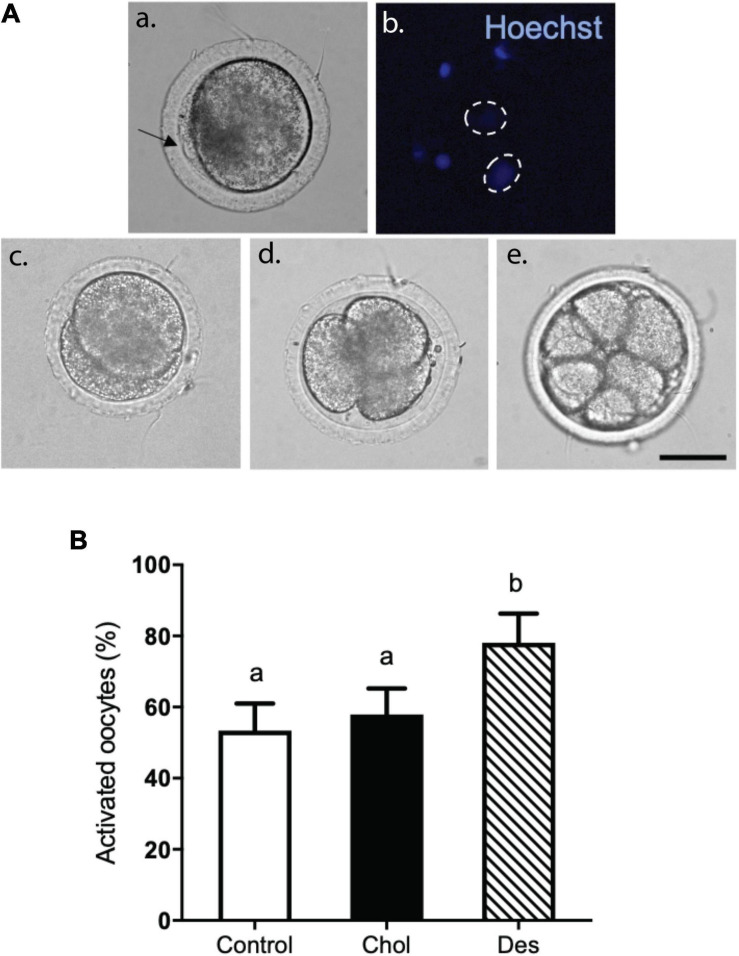
Effect of sterol incorporation on the ability of cryopreserved ram sperm to activate bovine oocytes *in vitro*. **(A)** Different hybrid cleavage stages of activated bovine oocytes after heterologous IVF with cryopreserved ram sperm pretreated with 10 mM MβCD-Chol (Chol) or -Des (Des). After co-incubation with sperm, oocytes were fixed and stained with Hoechst 33342. **(a)** Bright field of inseminated oocyte. The arrow shows polar bodies and adhered sperm to *zona pellucida*. **(b)** Oocyte *pronuclei* stained with Hoechst 33342 denoted by dashed circles and hybrid embryos with two **(c)** three **(d)** and eight cells **(e)**. Scale bar: 50μm. **(B)** Activation rate after heterologous IVF. Results are shown as percentages of activated oocytes and represent mean values ± SEM of 5 independent experiments from a total of 205 control oocytes, 199 MβCD-Chol treated oocytes and 189 MβCD-Des treated oocytes. Data were analyzed using Generalized Linear Mixed Models (GLMM) with Binomial family. The different letters (a, b) indicate significant differences (*p* < 0.05).

The fertilizing competence and ability of MβCD-sterol treated sperm to undergo capacitation and acrosome reaction physiologically after thawing was evaluated in heterologous IVF assays. After 48 h of *in vitro* culture, we evaluated the presence of *pronuclei* or oocyte cleavage as a consequence of oocyte activation by sperm (Figure 6). Developmental progress of oocytes that activated the cell division machinery resulted in asynchronous divisions and different stages of hybrid embryos (Figures 6Ac–e). Heterologous IVF with frozen-thawed ram sperm that incorporated desmosterol prior to freezing generated a higher percentage (>30%) of activated oocytes compared to the control and spermatozoa that incorporated cholesterol (Figure 6B). No statistical difference was observed between sperm treated with MβCD-Chol compared to the control.

### *In vivo* Fertility Outcomes

To evaluate if incorporation of cholesterol or desmosterol prior to cryopreservation increases fertility competence of frozen-thawed ram sperm *in vivo*, we performed a fertility trial by cervical AI of ewes during the breeding season. No differences were found in pregnancy rates of ewes inseminated with cryopreserved ram sperm from the different experimental groups (Table 2). However, ewes inseminated with sperm that incorporated desmosterol prior to cryopreservation resulted in significantly higher rates of live born lambs related to pregnant ewes when compared to the control and Chol group, indicative of reduced pregnancy loss or embryonic mortality in this experimental group.

**TABLE 2:** *In vivo* fertility of frozen-thawed ram sperm pretreated with 10 mM MβCD-Chol (Chol) or 10 mM MβCD-Des (Des) after cervical AI.

Experimental groups	Inseminated ewes N	Pregnancy rate N (%)	Live born/Inseminated N (%)	Live born/Pregnant N (%)	Pregnancy loss N (%)
Control	49	24 (48.9)	17 (34.7)	17 (70.8)	7 (29.2)
Chol	52	22 (42.0)	13 (25.0)	13 (59.1)	9 (40.9)
Des	59	24 (40.7)	23 (39.0)	23 (95.8) **	1 (4.2)

Pregnancy and birth rates were registered after cervical AI of 160 Texel ewes with frozen-thawed ram sperm. Pregnancy was detected at day 30 after insemination by ultrasonography. Data was analyzed by Chi- Square test for homogeneity. No differences were found in pregnancy rates (p = 0.68) or birth rates (p = 0.28) among the experimental groups. Statistically significant differences were found when live births relative to pregnancy were compared (p = 0.01). In particular, significant differences were found for the following comparisons: Control vs. Des (**p = 0.02) and Chol vs. Des (**p = 0.002). No differences were found for Control vs. Chol (p = 0. 40). Pregnancy loss is the counterpart of Live born/Pregnant.

## Discussion

Recently, we provided mechanistic insight into the differential role of cholesterol and desmosterol in ram sperm membrane and we proposed a cryopreservation procedure compatible with a standard protocol employing a sterol-free semen extender (Carro et al., 2020). The present study is focused on determining how treatment of ram sperm with MβCD-sterols prior to freezing influences sperm functionality and fertilizing competence *in vitro* and *in vivo*. Under these experimental conditions, sperm that incorporated cholesterol prior to freezing exhibited moderately higher levels of total sterols after thawing compared to the control. On the other hand, sperm that incorporated desmosterol showed intermediate levels of total sterols, not differing from the control or the cholesterol group. Conversely, direct fluorescence of BPY-Chol to estimate relative levels of sterols showed intermediate fluorescence intensity in MβCD-cholesterol treated sperm and moderately higher BPY-Chol associated fluorescence in MβCD-desmosterol treated sperm with respect to the control. These differences are not relevant in magnitude and reflect that total sterol content after freeze-thawing in sperm that have incorporated cholesterol or desmosterol before freezing is marginally higher with respect to the control. Prior to freezing, sperm almost double the original sterol level after MβCD-sterol treatment, irrespective of the sterol used in the complex (Chol or Des) (Carro et al., 2020). Considering the relative proportions of sterols prior to freezing, it can be inferred that desmosterol decreased from ∼50% (prior to freezing) to 35% (after thawing). Sperm preloaded with desmosterol showed a significantly different sterol composition (still different after thawing) compared to the control or cholesterol group. It can be also assumed that loss of sterols induced by cryopreservation was higher in treated groups compared to the control and even more interestingly, desmosterol content of Des group seems to have been selectively reduced over cholesterol. Higher loss of sterols after freezing and thawing was also observed by others in ram sperm, when adding cholesterol (Mocé et al., 2010b). Altogether, these results indicate that the mechanism underlaying sterol loss induced by freeze-thawing likely involves actual loss of sperm membrane, also evidenced in the decrease of major components of membrane bilayers after cryopreservation such as phospholipids (Chakrabarty et al., 2007).

BPY-Chol fluorescence also revealed the presence of different co-existing cell sub-populations, compatible with different sterol contents (Hölttä-Vuori et al., 2016; Bernecic et al., 2019; Carro et al., 2020). Previously, fresh sperm from control and 10 mM MβCD-sterol treated groups evidenced 4–6 different sperm sub-populations (Carro et al., 2020). Here we show that all experimental groups displayed no more than 3 sperm sub-populations after freeze-thawing, suggesting that there is a selective reduction in sub-population quantity caused by freezing. Notably, sperm categories with low fluorescence intensity (low sterol content) were increased after cryopreservation, which was reflected in a modal value different from that reported in fresh sperm, particularly in sperm treated with MβCD-sterol. In these experimental groups, a single sub-population comprised ∼60% of the sperm cells after freeze-thawing. The control also evidenced a sub-population which comprised 60% of the sperm cells but these cells showed low fluorescence, compatible with a low sterol content. Sperm sub-populations were previously described regarding sperm morphology (Thurston et al., 1999), osmotic tolerance (Druart et al., 2009), *in vitro* capacitation ability (Gadella and Harrison, 2000), and *in vivo* acrosome reaction competence (La Spina et al., 2016). Interestingly, in rams with proven *in vivo* fertility, distribution of motility-based sub-populations varied between high and low fertility rams (Ledesma et al., 2017). Moreover, it was reported that cryopreservation reduces the number of motility-based sub-populations in fish sperm (Gallego et al., 2017). Findings from our study draw on evidence of the close relation between sterol content and sensitivity of sperm to cryopreservation and also highlight the different sensitivity of sperm sub-populations based on the sterol content.

Cryopreservation causes severe damage to ram sperm structure and functionality, compromising its ability to reach and fertilize the oocyte. The role of cholesterol in mammalian sperm capacitation is well-known. High cholesterol level in the sperm membrane prevents premature capacitation and acrosome exocytosis, while sterol efflux triggers capacitation and associated post-testicular sperm maturation in the female tract (Gadella and Leahy, 2015; Cohen et al., 2016). Cholesterol loss from sperm membranes caused by freeze-thawing induces a premature capacitation-like state in the sperm (Cormier et al., 1997; Mocé et al., 2010a). Moreover, it has been reported that freezing and thawing triggers tyrosine phosphorylation in boar and stallion sperm (Green and Watson, 2001; Pommer et al., 2003). In our study, sperm that incorporated cholesterol maintained lower tyrosine phosphorylation levels after cryopreservation compared to control sperm and those that incorporated desmosterol. On the other hand, it has been reported that both cholesterol and desmosterol have the ability to prevent induction of human sperm capacitation *in vitro*, given by the planar structure of these sterols (Nimmo and Cross, 2003). Inhibition of sperm capacitation signaling by cholesterol could depend on the original level of this lipid at the plasma membrane (Darin-Bennett and White, 1977; Davis, 1981) and, therefore, may function differently in sperm with higher cholesterol to phospholipid ratios such as human sperm. In addition, non-effect of desmosterol on sperm tyrosine phosphorylation levels after cryopreservation highlights the differences between physiological capacitation and capacitation-like changes induced by freezing and thawing. Interestingly, it was recently shown that markers related to capacitation appear only in specific sub-populations of frozen-thawed stallion spermatozoa (Ortega-Ferrusola et al., 2017). Even when desmosterol-treated sperm exhibited more cryocapacitation compared to cholesterol-treated sperm, it is possible that non-capacitated sperm with good fertilizing potential still remains in the desmosterol group after freeze-thawing. Although cholesterol and desmosterol exerted differential effects on premature capacitation-like events induced by cryopreservation, they similarly decreased the percentage of acrosome-reacted sperm after freezing and thawing. Both sterols exerted a stabilizing effect on the acrosome, a Golgi-derived vesicle depleted of sterols at the membrane. Altogether, these results evidence that loss of acrosomal integrity after cryopreservation is more related to a mechanical stress, instead of a physiological triggering of acrosomal exocytosis (Nagy et al., 2003).

*In vitro* fertility after cholesterol incorporation has been previously evaluated in bull (Purdy and Graham, 2004), camel (Crichton et al., 2016), horse (Spizziri et al., 2010) boar (Blanch et al., 2012), and ram sperm (Mocé et al., 2010b). In accordance with our results, these reports showed that cholesterol incorporation does not increase the percentage of activated oocytes after IVF. However, evaluation of fertility outcome of cryopreserved sperm after desmosterol incorporation has not been explored. Moraes et al. (2010) reported that desmosterol incorporation into bull sperm does not improve binding to zona pellucida in fixed oocytes. Interestingly, we show novel results regarding significant activation of oocytes by desmosterol-enriched sperm after heterologous IVF. Frozen-thawed spermatozoa that incorporated desmosterol before cryopreservation contain approximately five times more desmosterol than the sperm that incorporated cholesterol. Sterols are also major components of membrane rafts, small domains that compartmentalize cellular processes and actively participate in sperm capacitation and fertilization (Gadella et al., 2008; Watanabe and Kondoh, 2011). However, cholesterol and desmosterol differ in their affinity to partition into these ordered domains (Vainio et al., 2006). Previously, we showed a reduced tendency of desmosterol compared to cholesterol for segregating into ordered domains (Carro et al., 2020). Therefore, it is likely to expect that a significant level of desmosterol over cholesterol could impact on membrane-dependent processes such as gamete interaction and fusion. Further research is needed to address how increased levels of desmosterol could affect membrane lateral organization, membrane topology, fusogenicity, and/or fertilization signaling.

As to *in vivo* fertility study, the insemination pilot trial carried out under these experimental conditions produced similar pregnancy rates in all experimental groups. Increased seminal quality of desmosterol-enriched sperm to withstand osmotic tolerance after cryopreservation (Carro et al., 2020), or increased ability to activate oocytes *in vitro* was not reflected into higher pregnancy rates. It is important to note that IVF assays bypass the physical barriers encountered by sperm in the female reproductive tract including physiological sperm selection, and therefore do not necessarily correlate with *in vivo* trials. Selection of sperm sub-populations are also blurred *in vitro*. However, when the lambing rates related to pregnancy were analyzed, ewes inseminated with spermatozoa that had incorporated desmosterol prior to freezing showed a significant increase in maintaining pregnancy to term, without registering almost any pregnancy loss or embryo mortality. It is widely accepted that progesterone is required for maintaining pregnancy (Spencer et al., 2004). Interestingly, it has been shown that progesterone inhibits the enzyme that converts desmosterol to cholesterol (DHCR24) in tissues and cells that accumulate desmosterol (Jansen et al., 2013). Upcoming fertility trials in extensive breeding programs coped with molecular analysis will contribute to a more comprehensive discussion.

On the other hand, membrane desmosterol could have contributed to better preserve male gamete genomic integrity. Even when we did not found differences in apoptotic DNA fragmentation assessed by TUNEL-reaction, other alterations produced in the chromatin structure could have been prevented by membrane desmosterol. Moderately low levels of DNA fragmentation were previously found by acridine orange (∼4%) and TUNEL (6%) in frozen-thawed ram sperm (Nur et al., 2010). Recently, ∼2.5% of DNA fragmentation index based on acridine orange was reported for ram sperm after cryopreservation (Peris-Frau et al., 2021). This study revealed that DNA methylation and apoptosis only increased after certain incubation times under capacitation conditions in cryopreserved spermatozoa, probably as a result of an early response to oxidative stress. It has been shown that sperm DNA damage is related to detrimental embryonic and/or fetal development in several species such as the bull (De Castro et al., 2016), fish (Pérez-Cerezales et al., 2010) and humans (Simon et al., 2014; Sedó et al., 2017). Moreover, epigenetic defects of the sperm could induce decreased developmental capacity of the generated embryos, providing a possible explanation for early pregnancy loss (Stuppia et al., 2015). Considering the growing evidence on male contribution to embryo development and involvement of gene regulators such as histone modifications and small non-coding RNAs (Baxter and Drake, 2019; Le Blévec et al., 2020), this emerging field of study deserves further research in domestic species, particularly in relation to biotechnologies applied for increasing reproductive efficiency.

Taken together, findings from our study reveal differential effects of cholesterol and desmosterol incorporation into ram sperm on functional parameters and fertility competence after cryopreservation. First, a different sterol composition characterized pre-treated sperm after freeze-thawing. Interestingly, sterol-based sperm sub-populations were differentially affected by freeze-thawing. Cholesterol incorporation reduced tyrosine phosphorylation levels mitigating the effects of cryopreservation on the induction of capacitation-like events. However, both sterols decreased damage caused by freeze-thawing to acrosome membranes. In this regard, treatment did not reduce the ability of sperm to undergo capacitation and acrosome reaction after cryopreservation, as shown here by IVF assay. Finally, we show that desmosterol-enriched sperm give rise to higher rates of *in vitro* activated oocytes by heterologous fertilization and to significantly lower pregnancy loss *in vivo*. These results are particularly important considering that increase of sperm desmosterol content has not been studied as a cryoprotective strategy. Our research provides new insights into desmosterol positive influence in ram sperm fertility. Moreover, our work opens a novel and challenging research subject aimed at elucidating biochemical and cellular processes involved in this protective mechanism. Deciphering epigenetic contribution of desmosterol-protective effect should be the focus of future studies.

## Data Availability Statement

The original contributions presented in the study are included in the article, further inquiries can be directed to the corresponding author/s.

## Ethics Statement

The animal study was reviewed and approved by the Institutional Committee for the Care and Use of Experimental Animals (CICUAE-Resolution 046/2016).

## Author Contributions

MC: conceptualization, methodology, software, formal analysis, investigation, and writing—original draft. RR-V and DP: conceptualization, methodology, formal analysis, and investigation. JB: conceptualization, methodology, formal analysis, resources, writing-original draft, writing—review and editing, supervision, and funding acquisition. FH: conceptualization, investigation, resources, writing—review and editing, project administration, and funding acquisition. All authors contributed to the article and approved the submitted version.

## Conflict of Interest

The authors declare that the research was conducted in the absence of any commercial or financial relationships that could be construed as a potential conflict of interest.
